# Young nursing and medical students’ knowledge and attitudes towards sexuality and contraception in two spanish universities: an inferential study

**DOI:** 10.1186/s12909-023-04255-8

**Published:** 2023-04-26

**Authors:** Juan-Pablo Scarano-Pereira, Alessandro Martinino, Francesca Manicone, Cristina Álvarez-García, Lucía Ortega-Donaire, María-Zoraida Clavijo-Chamorro, Isabel M López-Medina, Carmen Álvarez-Nieto, Sebastián Sanz-Martos

**Affiliations:** 1 Faculty of Medicine, Complutense University of Madrid, Madrid, Spain; 2 Faculty of Medicine and Dentistry, La Sapienza University of Rome, Rome, Italy; 3 Department of Nursing, Faculty of Health Sciences, University of Jaen, Edif. B3, dep. 243, Campus Las Lagunillas, s/n, Jaén, 23071 Spain; 4 Department of Nursing, University of Extremadura, Extremadura, Spain

**Keywords:** Contraception, Unwanted pregnancy, Primary prevention, Knowledge, Young adult

## Abstract

**Background:**

Living safely sexuality and without risk to one’s health is an international priority. The youth age group has specific characteristics that make it a particularly vulnerable group for adverse consequences such as unwanted pregnancies or sexually transmitted infections. Health professionals are an important group to address this issue; however, to achieve a good result, sufficient knowledge is required to solve all the issues. This study aimed to assess the level of knowledge of young university students studying a nursing or a medical degree.

**Methods:**

A descriptive cross-sectional study of young medical and nursing students was conducted. The selection of participants was made by convenience. The Sexuality and Contraceptive Knowledge Instrument scale was used to measure knowledge level. A bivariate analysis was conducted using the Mann–Whitney U test or the Kruskal–Wallis H test, depending on the number of categories of the independent variable. Finally, a multivariate analysis was conducted using a multiple linear regression model, establishing the level of knowledge as the dependent variable and all variables that obtained statistical significance in the bivariate analysis as predictors. Data collection was carried out from October 2020 to March 2021.

**Results:**

The sample comprised 657 health university students. Participants had a good level of knowledge, with 77.9% answering 50% of the questions correctly. Before training, 34.15% of the participants did not pass 50% of the questions asked. This percentage decreased to 12.87% after receiving sexuality training during their university degrees. The main training gaps were found for the items on hormonal contraceptive methods. The bivariate analysis showed that female participants had significantly higher knowledge scores, as did those who had used a hormonal contraceptive method during the most recent intercourse or were aware of family planning centers. These variables maintained their significant effect at the multivariate level, obtaining two models with good explanatory power for participants of both university degrees.

**Conclusion:**

The general level of knowledge of the healthcare students was high and sufficient after receiving training during the university degree (87.13% of the participants obtain more than 50% of items correct). The main training gap was found for items on hormonal contraceptive methods, which should be emphasized in future training programs.

**Supplementary Information:**

The online version contains supplementary material available at 10.1186/s12909-023-04255-8.

## Background

Sexual health can be defined as a state of physical, emotional, mental, and social well-being in the field of sexuality and is recognized as a global priority by the World Health Organization [1]. Related to the concept of sexual health is the concept of sexuality as a fundamental dimension of the human being, which becomes more evident from the biological and socio-cultural changes derived from puberty in adolescence. At this stage, adolescents and young people may be physically ready to begin their sexual life but may not have the psychological and conceptual preparation to begin it safely [2].

The youth age group of 18–25 is considered at particular risk for experiencing adverse consequences such as sexually transmitted infections, unwanted pregnancies, or abortions [3–6]. According to previous research, the highest prevalence of sexually transmitted infections is found in the under-25 age group, as well as the highest rate of voluntary termination of pregnancy [7–9]. These consequences may have repercussions in terms of academic and professional opportunities. One of the reasons given in previous researches for this high prevalence is the decrease in the age at first sexual intercourse, which may increase the period of sexual activity of young people and the total number of sexual partners. On the other hand, the psychological characteristics of adolescents and youth, such as invulnerability or false perception of risk, may make them particularly susceptible to adopting risky behaviors related to their sexuality [2, 5, 10, 11]. In Spain (in 2021), for the under 19 age group, the rate of voluntary termination of pregnancy was 7.90 cases per 1000 women, while for the 20–24 age group the rate was 16.09 events per 1000 women that is the highest of all age groups studied. [12]

In order to experience sexuality without associated risks, it is necessary to have a sufficient level of knowledge to develop positive attitudes toward the use of the different contraceptive options available. Health professionals are quality sources of information that young people can approach for information; however, access to healthcare by young people is often low. Moreover, youth show a high prevalence of using other sources of information that do not require two-way interaction, such as the internet. Other sources of information that were used previously, such as peer groups or parents, are now less prevalent [13, 14]. Family planning centers are an important option for the education of young people, where health professionals can provide quality training adapted to their needs. Previous researchers have found higher levels of knowledge, more positive attitudes toward the use of contraceptive methods, and a lower rate of sexually transmitted infections in young people who are aware of these centers and have used them [15–17].

Young healthcare students are a population vulnerable to risky sexual practices due to the characteristics of their age group. Hence, they need an adequate level of knowledge about sexuality and contraceptive methods to experience it safely. In addition, as future health professionals, they need to acquire the knowledge and skills to provide quality information in health care centers or family planning centers. Knowing the level of knowledge of the undergraduate students, before the training and after receiving it, during their university degree, we can obtain a diagnosis of the main gaps of the young population, guiding the future university programs and improving the training in the Higher Education. Therefore, this study aimed to assess the level of knowledge about sexuality and contraceptive methods of young students of healthcare degrees.

## Methods

### Participants and study modules

A descriptive cross-sectional study was carried out with undergraduate students of the Medical degree at the Complutense University of Madrid (Spain) and of the Nursing Degree at the University of Jaen (Spain), aged between 18 and 25 years. The sample size was calculated based on the results of previous researches [18, 19]; a sample size calculation was established to detect a difference of 1 point in knowledge scale, a standard deviation of 2.5 points, a confidence level of 95%, and a power of 80%. Therefore, the minimum sample size was established at 200 participants.

### Procedure

Data were collected by trained teachers from healthcare students studying a six-year medical degree and a four-year nursing degree, between October 2020 and March 2021. The sample was selected by convenience sampling among the nursing and medical students who attended to training sessions, where the information was collected. In order to complete the data collection document, 15 min of teaching classes were used, in which a researcher was always present. The self-administered data collection notebook in paper format included the following:

- Socio-demographic variables: Sex, Age, University, Academic year, Having received training on sexuality and contraceptive methods during the medical or Nursing degree, Having a partner at the time of the study, Have viewed pornographic material during adolescence, Currently viewing pornographic material, Perceived usefulness of using pornographic material as an educational element, Source of information used to obtain information on sexuality and contraceptive methods, Desired source of information to obtain information on sexuality and contraceptive methods, Self-perceived level of knowledge about sexuality and contraceptive methods, and Self-perceived knowledge gap.
- Level of knowledge about sexuality and contraceptive methods: Measured through the Sexuality and Contraceptive Knowledge Instrument scale, validated in Spanish and consisting of 15 items with three response options (True, False and Don’t know/No answer). The scale showed a reliability of 0.99 for the items and 0.74 for the persons; the intraclass correlation coefficient for test-retest reliability obtained a value of 0.81 [18]. The score range is between 0 and 15, and the level of knowledge is categorized as follows: excellent (≥ 90% correct), very good (89–70%), good (69–50%), and insufficient (< 50%) [19].
- Sexual intercourse variables: Ever had penetrative sex, Age at first intercourse, Use of contraceptive method at first intercourse, Contraceptive method used or reason for not using any, Use of contraceptive method at last intercourse, and Contraceptive method used or reason for not using any.
- Knowledge about Family Planning Centers (FPC): This was assessed by a dichotomous question (Yes/No). Participants who answered in the affirmative were asked to respond to 6 statements by indicating whether they considered them True, False or Don’t know/No answer. If the number of correct answers is above 50%, the student was considered to be aware of the FPCs. This section of the questionnaire was designed ad hoc and was peer-reviewed.

To achieve a faithful approximation to reality, a distinction was made between participants who had received specific training on sexuality and contraceptive methods during their university degree. For the nursing degree, the training was received in the second year and for the medical degree, it was during the fourth year.

### Data analysis

Descriptive statistics for all data and the questionnaire score were calculated. Bivariate analyses were performed, establishing as dependent variable the score on the knowledge scale, and as independent variables the socio-demographic variables, sexual relations variables, and the variables on knowledge of FCPs. All the bivariate analyses were differentiated by the university degree that the participants were studying as variables related to training were also analyzed. Moreover, the questionnaires were carried out in different academic years, and we could commit a confounding bias when making comparisons by the socio-demographic variables if joining both groups. The normality of the distribution was assessed by analyzing the histogram, skewness (0.081), kurtosis (-0.725), and the Kolmogorov-Smirnov test (KS = 0.102; P < 0.01). Contrasts were performed using the non-parametric Mann–Whitney U or Kruskal–Wallis H statistic depending on the number of categories of the independent variable.

A multivariate analysis was performed using a multiple linear regression model, establishing the level of knowledge as the dependent variable and those variables that obtained statistical significance at the bivariate level as predictor variables. The model`s goodness of fit was calculated using the value of R^2^. All analyses were performed with the statistical program SPSS 24.0, and a value of p < 0.05 was established as the level of statistical significance.

## Results

The initial sample consisted of 696 participants. Of these, 11 were excluded for not completing the questionnaire properly and 28 for being over 25 years of age. The final sample included 657 participants (303 from the Nursing Degree and 354 from the Medical degree), with a mean age of 20.65 (SD: 2.32) years. The main source of information used to learn about sexuality and contraceptive methods was the internet, followed by Healthcare professionals. The main sources of information requested by participants were Talks on sexual and reproductive health and digital resources on the internet with high-quality information. The leading information gap identified was for non-coital sexual relations, followed by information about contraceptive methods and where to obtain quality information. Just over half (55.4%) of the sample viewed pornographic material during adolescence, with a mean age of onset of 15.18 (SD: 2.42) years. Table 1 shows the demographic characteristics of the sample.

**Table 1:** Demographic characteristics of the sample

Variable	Category	Nursing	Medicine	Total
**Gender**	Men	65	71	136
	Women	238	283	521
**Age (years)**		19.75 (1.83)	21.42 (2.42)	20.65 (2.32)
**Academic year**	1st Year	80	63	-
	2nd Year	109	53	-
	3rd Year	24	46	-
	4th Year	90	28	-
	5th Year	-	99	-
	6th Year	-	65	-
**Received sexuality training during the university degree**	Yes	201	114	345
	No	102	210	312
**Has a partner**	Yes	186	197	383
	No	117	157	274
**Source of information used to obtain information on sexuality and contraceptive methods**	Internet	119	198	317
	Healthcare professionals	111	74	185
	Family	22	15	37
	Friends	51	67	118
**Source of information desired to obtain information on sexuality and contraceptive methods**	Talks on sexual and reproductive health	175	127	302
	Internet	122	222	344
	TV campaigns	6	5	11
**Pornography use during adolescence**	Yes	180	184	364
	No	123	170	293
**Age of use**		15.06 (2.29)	15.28 (2.53)	15.18 (2.42)
**Perception of the effectiveness of pornographic material**	Good	4	9	13
	Regular	20	31	51
	Bad	64	62	126
	Very bad	109	116	225
**Self-perception of their knowledge about sexuality and contraceptive methods**	Good	108	122	230
	Regular	102	124	226
	Bad	93	108	201
**Had penetrative sex**	Yes	239	259	498
	No	64	95	159
**Age at first intercourse**		16.40 (1.29)	17.35 (1.99)	16.89 (1.76)
**Use of any contraceptive method during first intercourse**	Yes	213	231	444
	No	26	28	54
*Contraceptive method used during first intercourse*	Male condom	206	207	413
	Hormonal contraceptive methods	5	23	28
	Withdrawal method	2	1	3
*Reason for not using any contraceptive method during first intercourse*	Improvised sexual intercourse	18	22	40
	Were not planning to use it	3	4	7
	Shame to get a contraceptive method	5	-	5
	Alcohol consumption	-	2	2
**Use of any contraceptive method during most recent intercourse**	Yes	201	227	428
	No	38	32	70
*Contraceptive method used during the most recent intercourse*	Male condom	136	146	282
	Hormonal contraceptive methods	62	78	140
	Withdrawal method	3	3	6
*Reason for not using any contraceptive method during most recent intercourse*	Improvised sexual intercourse	10	11	21
	Reduce pleasure	10	6	16
	Were not planning to use it	18	13	31
	I have a permanent partner	-	2	2
**Knowledge about FPCs**	Yes	99	129	228
	No	204	225	429

FPCs: Family planning centers

Data expressed as absolute frequencies except Age (Mean and SD)

The scale showed acceptable reliability values for the total sample (α = 0.715) and for the subsamples of medical students (α = 0.715) and nursing students (α = 0.705). The average score for the knowledge scale was 8.73 (2.87). Medical students had a significantly higher score (9.14 [2.81] vs. 8.58 [2.893]; p = 0.011). Regardless of the academic degree they studied,77.9% of the participants obtained a knowledge level value rated as good, very good, or excellent. Before receiving the relevant sexuality education in either of the university degrees, 34.15% of the participants obtained “insufficient” knowledge, a percentage that decreased to 12.87% after receiving this training during their degree; this difference was statistically significant (p < 0.05). The items with the highest percentage of lack of knowledge were the items on hormonal contraceptive methods such as the contraceptive pill, vaginal ring or contraceptive patch, regardless of the academic level of the participants, reaching an error rate and lack of knowledge of 54.57%. The items on using the male condom or general sexuality concepts obtained a percentage of correct answers close to 90% in both groups.

In the samples of both university students, we found similar results at the bivariate level, finding common significant variables such as female sex, having a partner at the time of the study, having penetrative sex prior to the time of the study, using a hormonal contraceptive method during the most recent intercourse, and know about the FPCs. With respect to age, we found a moderate significant positive correlation in both samples, indicating that older participants at the time of the study had a higher level of knowledge. Table 2 shows the bivariate contrasts for both university grades.

**Table 2:** Bivariate contrasts

Variable	Nursing	Contrast	Medicine	Contrast
**Gender**				
Men	7.48 ± 2.87	*Z* = − 2.563*	8.01 ± 2.84	*Z* = − 3.779**
Women	8.26 ± 2.82		9.42 ± 2.73	
**Age**		*Rho* = 0.340**		*Rho* = 0.524**
**Academic course**				
1st Year	6.51 ± 2.07	*χ*^*2*^ = 77.833**	7.08 ± 2.07	*χ*^*2*^ = 117.512**
2nd Year	10.11 ± 2.23		7.11 ± 2.07	
3rd Year	9.25 ± 2.52		8.52 ± 2.48	
4th Year	8.40 ± 2.3.23		10.14 ± 2.43	
5th Year			10.37 ± 2.73	
6th Year			10.91 ± 1.93	
**Received sexuality training during the university degree**				
Yes	8.79 ± 2.86	*Z* = − 4.587**	10.41 ± 2.47	*Z* = − 8.628
No	7.22 ± 2.42		7.93 ± 2.57	
**Source of information used to obtain information on sexuality and contraceptive methods**				
Internet	7.82 ± 2.80	*χ*^*2*^ = 26.739**	9.04 ± 2.85	*χ*^*2*^ = 15.227**
Healthcare professionals	9.30 ± 2.65		10.12 ± 2.67	
Family	7.53 ± 2.89		8.63 ± 2.52	
Friends	7.14 ± 2.12		7.87 ± 3.04	
**Has a partner**				
Yes	8.51 ± 2.82	*Z* = − 1.982*	9.74 ± 2.60	*Z* = − 4.383**
No	7.86 ± 2.80		8.39 ± 2.88	
**Pornography use during adolescence**				
Yes	8.14 ± 2.82	*Z*= -1.054	8.98 ± 2.95	*Z* = − 1.238
No	8.44 ± 2.86		9.31 ± 2.65	
**Self-perception of their knowledge about sexuality and contraceptive methods**				
Good	8.43 ± 2.85	*χ*^2^ = 2.148	9.38 ± 2.82	*χ*^*2*^ = 1.421
Regular	8.41 ± 2.86		9.17 ± 2.70	
Bad	7.90 ± 2.75		8.83 ± 2.82	
**Had penetrative sex**				
Yes	8.48 ± 2.82	*Z* = − 2.685**	9.83 ± 2.68	*Z* = − 7.648**
No	7.44 ± 2.72		7.25 ± 2.21	
**Age at first intercourse**		*Rho* = − 0.073		*Rho*= -0.030
**Use of any contraceptive method during first intercourse**				
Yes	8.41 ± 2.79	*Z* = − 1.305	9.84 ± 2.73	*Z* = − 0.145
No	9.08 ± 3.01		9.71 ± 2.34	
**Contraceptive method used during first intercourse**				
Male condom	8.35 ± 2.78	*χ*^*2*^ = 4.089	9.77 ± 2.78	*χ*^*2*^ = 2.348
Hormonal contraceptive methods	11 ± 2.55		10.57 ± 2.19	
Withdrawal method	7.5 ± 0.71		8 ± 0	
**Use of any contraceptive method during most recent intercourse**				
Yes	8.52 ± 2.84	*Z* = − 0.467	9.89 ± 2.60	*Z* = − 0.652
No	8.26 ± 2.69		9.41 ± 3.22	
**Contraceptive method used during the most recent intercourse**				
Male condom	8.03 ± 2.93	*χ*^2^ = 15.402**	9.54 ± 2.63	*χ*^2^ = 6.685*
Hormonal contraceptive methods	9.66 ± 2.30		10.55 ± 2.45	
Withdrawal method	7.33 ± 3.22		9.67 ± 2.08	
**Knowledge about FPCs**				
Yes	8.95 ± 2.78	*Z* = − 2.710**	10.40 ± 2.69	*Z* = − 6.304**
No	7.98 ± 2.79		8.46 ± 2.63	

FPCs, Family planning centers

*P < 0.05; **P < 0.01;

Rho = Spearmean Rho; Z: Contast with Mann–Whitney U; *χ*^2^: Contrast with Kruskal–Wallis H

Eight variables were introduced into the model for the **Medical degree**. The model consisted of 4 predictors, with a high correlation among them and the knowledge scale score (0.574), and has an explanatory power of 31.8% of the variance. The ANOVA for the multiple regression model showed a statistically significant relationship F = 27.318; p < 0.001. The collinearity of the model by the model conditioning index obtained a value of 17.034, considered moderate collinearity. The Durbin Watson statistic obtained a value of 2.113, so the residuals were considered independent.

For the **Nursing degree**, 8 variables were also introduced into the model, but the age variable was excluded because of collinearity problems with the rest of the variables. The values of the model were calculated with the remaining 7 variables, obtaining a final model composed of 5 predictor variables. The variables of the final model presented a moderate correlation value (0.497), with an explanatory capacity of 22.7%. The ANOVA for the multiple regression model also showed a statistically significant relationship F = 9.115; p < 0.001. Collinearity was also moderate (16.056), and the Durbin Watson statistic was 1.763; therefore, the residuals were considered independent. Table 3 shows the values of the predictor variables for both models.

**Table 3:** Model values for the scale of knowledge about sexuality and contraceptive in nursing and medicine degree students

Medical degree			
Variable	Beta	*p*	Correlation coefficient
Academic year	0.460	< 0.001	0.486
Gender	0.195	0.001	0.229
Knowledge about FPCs	0.165	0.004	0.193
Contraceptive method used during the most recent intercourse	0.114	0.040	0.137
**Nursing degree**			
**Variable**	**Beta**	***p***	**Correlation coefficient**
Received training during the university degree	0.504	< 0.001	0.395
Academic year	-0.385	< 0.001	-0.131
Contraceptive method used during the most recent intercourse	0.205	0.001	0.227
Knowledge about FPCs	0.145	0.024	0.160
Gender	0.131	0.036	0.149

FPCs: Family planning centers

Beta: Correlation value between predictor variable and knowledge level

### Discussion

The education of healthcare students on sexuality and contraception is a key element in ensuring that they are reliable sources of health information in the future. The level of knowledge of all participants in our study was good after they had received specialized training as part of their university training. Our result contrasts with previous research on the level of knowledge of university healthcare students [17, 20], where a low level of knowledge was found. These differences may be because the study by Htay et al. [17] evaluated students during their last year of medical school. However, our data reveal that there is a slight decrease in the years following sexuality training due to a loss of knowledge over time, although this was only evident in the Nursing degree students. Therefore, the effect of the participant’s age and the time at which sexuality training on the subject took place should be considered.

The variability in the level of knowledge between the two degrees may be related to the teaching methodologies used. An educational intervention carried out at the University of Jaén, where students of the Nursing degree were trained using a peer education methodology, which incorporated a clinical simulation activity using role play for different clinical scenarios, found a statistically significant improvement in the level of knowledge following the educational intervention, and an improvement in attitudes toward using contraception, with more positive attitudes among participants who had used a contraceptive method in their last sexual intercourse [19]. This experience highlights the need to modify the teaching methodologies used for sexuality education from a top-down methodology, where a trained teacher delivers content to a passive audience, to a participatory methodology that engages participants in an active role. Incorporating a more active and dynamic education on sexuality and contraception for young people can help break down or remove the barrier of addressing a taboo subject by encouraging open conversation about an individual’s concerns.

Health professionals may present some barriers to training on this topic, as Blakey and Aveyard [21] found in their systematic review, where they show that professionals lack models or training structures on how sexuality should be addressed to train young people. Another barrier they identified was that health professionals perceived their knowledge of sexuality and contraception to be low, which is in line with our research [21]. Only one-third of our sample perceived their level of knowledge as good, which is not consistent with the level of knowledge presented in the numerical rating. This may indicate a low self-perception of knowledge level and highlights a need to develop activities to build up not only conceptual attitudes but also communicative attitudes that allow them to approach the subject with greater confidence.

The level of knowledge was significantly higher in female participants in both groups, similar to previous research, where women showed better results in questions not related to knowledge of the male condom [22, 23]. In the present study, the primary educational gaps in both groups were found in items related to the use of hormonal contraceptive methods, which may explain this difference, similar to a previous research with nursing students from 10 Spanish universities [15]. Another possible explanation may be due to the differential impact that an unwanted pregnancy may have during the formative stage, which may mean that women are more likely to have a greater predisposition to acquire the knowledge to prevent this [24].

An aspect shown in previous research that we have also found in our study is the importance of FPCs in addressing the issue of sexuality and contraceptive education. This result highlights the importance of obtaining information from quality sources such as health professionals in these centers [15, 25]. Related to this difference in the level of knowledge, we highlight the finding that those who used some type of hormonal contraceptive method during the most recent intercourse obtained higher scores on the scale. This may be explained by a difference in knowledge according to sex, where women (being the ones who are going to use this contraceptive method) have received more in-depth education regarding this method [22, 23, 26, 27]. Alternatively, due to these contraceptive methods requiring a medical prescription, women have obtained information through a health professional, while young people who used the male condom, which is over-the-counter, did not need to talk to a health professional to obtain it [28].

To achieve a risk-free experience of sexuality and an increase in the rate of contraceptive use, we need to look at it from a gender perspective and gain a deeper understanding of the perceptions that may lead to non-use of contraception. Our research asked about the main reasons for not using any contraceptive method in previous sexual relations. We found aspects related to the use of the male condom and the sexual practice itself—“they take away pleasure”—and related to the unpredictability of sexual relations and the associated adrenergic discharge—“sexual relations were improvised”—; results, similar to previous research, where only the reasons for not using the male condom were addressed [29]. This result highlights the importance of promoting contraceptive options other than the male condom through future training strategies.

From our research findings on the primary sources of information used by young people, we found an increasing demand for digital media. In fact, the internet was the main source of information, a finding consistent with previous research [13, 14], which leads us to highlight the importance of creating a mobile application where quality content can be accessed. This content should include information about the main face-to-face resources to access qualified health professionals based on their access preferences, and in this way, improve the access of this population group to the relevant quality education [30].

As a result of the research, two regression models were obtained for the level of knowledge, finding common variables such as female gender, use of a hormonal contraceptive method and knowledge about family planning centers, similar to previous multicenter research [15]. This increases the reliability of the results of our research, showing a variable of majority training in women through family planning centers.

Among the possible limitations of our study, we highlight that as a voluntary survey, the motivation of participants to participate may affect the results. The level of knowledge measured may be affected by this motivation, and the selected population segment may cause an overestimation or underestimation of the value analyzed. On the other hand, being a taboo subject, participants may not fully disclose their reality about previous sexual relations, which may cause the rate of contraceptive use in previous sexual relations reported by participants to be overestimated. We should be cautious when extrapolating the results of this research, since the sampling method was non-probabilistic (by convenience). As a strength of our study, we highlight that it is a novel study describing the main gaps in knowledge found in young nursing and medical students, which could serve as a basis for developing new training strategies to improve the education of future health professionals.

## Conclusion

The general level of knowledge of the healthcare degree students was high and sufficient to be able to respond as future health professionals to the demands placed on them, both at a personal and professional level. The main training gap we found in both groups was for hormonal contraceptive methods. This aspect should be emphasized in future training programs, without ignoring the gender gap found in the level of knowledge. One aspect to highlight when providing training is the incorporation of active-participatory teaching methodologies that allow transferring theoretical concepts to common practical aspects, improving the level of knowledge, attitudes towards contraceptive options and self-perception of their training as health professionals, achieving a greater predisposition to meet health demands and a better approach.

Further research is needed on behavioral patterns among both sexes that may affect the level of knowledge and use of different contraceptive options in future sexual relations.

## Electronic supplementary material

Below is the link to the electronic supplementary material.

Supplementary Material 1

## Data Availability

All data generated or analysed during this study are included in additional file 1.

## List of Abbreviations

SD: Standard Deviation
FPCs: Family Planning Centers
ANOVA: Analysis of Variance
